# Supplementary material for: Establishment and Characterization of SV40 T-Antigen Immortalized Porcine Muscle Satellite Cell
Source: Cells. 2024 Apr 18;13(8):703. doi: 10.3390/cells13080703 (PMC11049531; doi:10.3390/cells13080703)
Supplement: Supplementary file 1 [file cells-13-00703-s001.zip › cells-2941368-supplementary.pdf]

Supplementary Information

Establishment and Characterization of SV40 T-Antigen Immortalized Porcine Muscle Satellite Cell

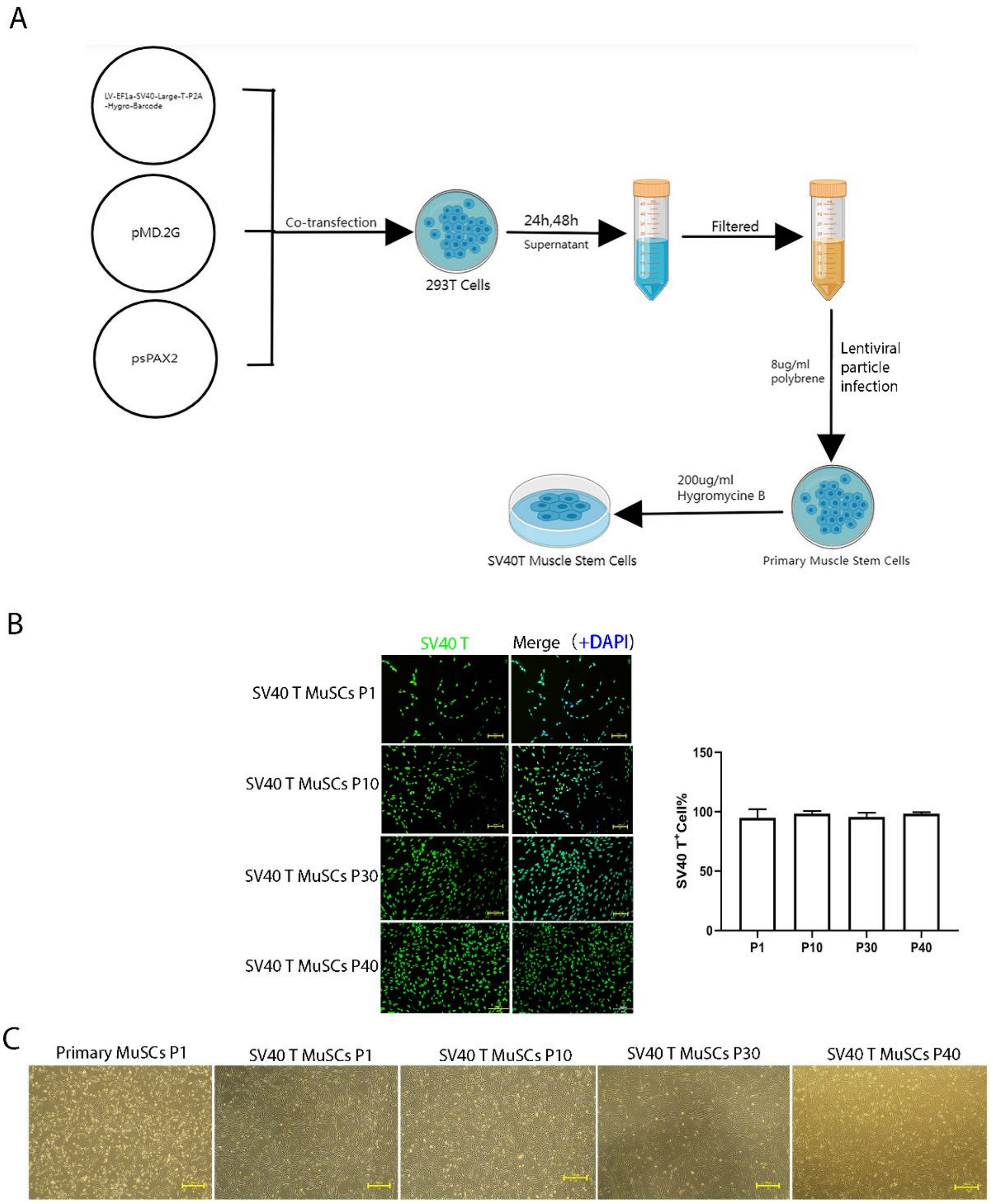


Supplementary Figure S1 ( Related to Figure 3): Establishment and characterization of SV40 T-pMuSCs. (A) Flow chart of lentivirus infection in primary MuSCs. (B) Immunostaining of SV40T MuSCs at P1, P10, P30, and P40 generations using DAPI (blue) and anti-SV40T (green) antibody. Scale bar, 100 µm. (C) Morphological images of P1-generation primaryMuSCs and SV40T pMuSCs at different passages.
